# Supplementary material for: From sequence to enzyme mechanism using multi-label machine learning
Source: BMC Bioinformatics. 2014 May 19;15:150. doi: 10.1186/1471-2105-15-150 (PMC4229970; doi:10.1186/1471-2105-15-150)
Supplement: Additional file 2 — Java code of ml2db. Additional file ml2db_code.tar.gz contains the Java source code to run the multi-label machine learning experiments and save the results to database. The code’s Javadoc is included. [file 1471-2105-15-150-S2.zip › additional file 2/ml2db/utils/doc/uk/ac/ed/inf/utils/setutils/SetUtils.html]

SetUtils


---


|  |  |  |
| --- | --- | --- |
| **Overview** \| **Package** \| **Class** \| **Use** \| **Tree** \| **Deprecated** \| **Index** \| **Help** | |  |
| **PREV CLASS**   **NEXT CLASS** | **FRAMES**    **NO FRAMES**     **All Classes** |  |
| SUMMARY: NESTED \| FIELD \| CONSTR \| METHOD | DETAIL: FIELD \| CONSTR \| METHOD |  |


---


## uk.ac.ed.inf.utils.setutils Class SetUtils

```
java.lang.Object
  uk.ac.ed.inf.utils.setutils.SetUtils
```

---

``` public class SetUtils extends java.lang.Object ```

Class

**Version:**
:   14 Mar 2011

**Author:**
:   Luna De Ferrari luna.deferrari-at-ed.ac.uk

---

**Constructor Summary**

`SetUtils()`


| **Method Summary** | |
| --- | --- |
| `static java.util.TreeSet<java.lang.String>` | `fromArrayToSet(java.lang.String[] array)` |
| `static java.util.TreeSet<java.lang.String>` | `fromStringToSet(java.lang.String string, java.lang.String divider)` |
| `static java.lang.String[]` | `fromTreeSetWithNullsToArray(java.util.TreeSet<java.lang.String> set)` |
| `static java.lang.Double` | `getHighestValue(java.util.TreeSet<java.lang.Double> set)` |
| `static boolean` | `isSubsetOfSuperset(java.util.TreeSet<java.lang.String> subset, java.util.TreeSet<java.lang.String> superset)` |
| `static java.lang.String` | `setToString(java.util.TreeSet<java.lang.String> set)` |

**Methods inherited from class java.lang.Object**

`equals, getClass, hashCode, notify, notifyAll, toString, wait, wait, wait`

**Constructor Detail**

### SetUtils

```
public SetUtils()
```


**Method Detail**

### fromArrayToSet

```
public static java.util.TreeSet<java.lang.String> fromArrayToSet(java.lang.String[] array)
```

---


### fromStringToSet

```
public static java.util.TreeSet<java.lang.String> fromStringToSet(java.lang.String string,
                                                                  java.lang.String divider)
```

---


### fromTreeSetWithNullsToArray

```
public static java.lang.String[] fromTreeSetWithNullsToArray(java.util.TreeSet<java.lang.String> set)
```

---


### getHighestValue

```
public static java.lang.Double getHighestValue(java.util.TreeSet<java.lang.Double> set)
```

---


### isSubsetOfSuperset

```
public static boolean isSubsetOfSuperset(java.util.TreeSet<java.lang.String> subset,
                                         java.util.TreeSet<java.lang.String> superset)
```

---


### setToString

```
public static java.lang.String setToString(java.util.TreeSet<java.lang.String> set)
```


---


|  |  |  |
| --- | --- | --- |
| **Overview** \| **Package** \| **Class** \| **Use** \| **Tree** \| **Deprecated** \| **Index** \| **Help** | |  |
| **PREV CLASS**   **NEXT CLASS** | **FRAMES**    **NO FRAMES**     **All Classes** |  |
| SUMMARY: NESTED \| FIELD \| CONSTR \| METHOD | DETAIL: FIELD \| CONSTR \| METHOD |  |


---
